# LIGHT Amplification by NF-*κ*B Contributes to TLR3 Signaling Pathway-Induced Acute Hepatitis

**DOI:** 10.1155/2023/3732315

**Published:** 2023-01-09

**Authors:** Dongming Lai, Zejian Lv, Xiaohua Lu, Peng Shang, Yunyun Geng, Pu Wang

**Affiliations:** ^1^Sun Yat-sen Memorial Hospital, Sun Yat-sen University, Guangzhou 510120, China; ^2^Guangdong Provincial People's Hospital & Guangdong Academy of Medical Sciences, Guangzhou, Guangdong 510080, China; ^3^Shenzhen Institutes of Advanced Technology, Chinese Academy of Sciences, Shenzhen 518055, China; ^4^Hebei University of Chinese Medicine and Heibei Key Laboratory of Chinese Medicine Research on Cardiocerebrovascular Disease, Shijiazhuang, Hebei 050000, China; ^5^Cytovaxis Biotechnologies Inc, Guangzhou, Guangdong 510760, China

## Abstract

LIGHT is a member of the TNF superfamily and a proinflammatory cytokine involved in liver pathogenesis. Many liver diseases involve activation of Toll-like receptor 3 (TLR3), which is activated by double-stranded RNA (dsRNA). However, the involvement of LIGHT in TLR3 implicated liver diseases is not clear. In this study, we investigated the role of LIGHT in TLR3 involved liver pathogenesis by using a mouse model of TLR3 agonist poly(I:C)-induced hepatitis. We found LIGHT expression at both protein and mRNA level in liver tissues is dramatically increased during the course of poly(I:C)-induced liver injury. This induction depends on NF-*κ*B activation as pretreating the mice with a NF-*κ*B inhibitor abrogates LIGHT upregulation. Importantly, blockade of the LIGHT signaling pathway with the recombinant LIGHT receptor HVEM protein ameliorates liver injury in poly(I:C)-induced hepatitis. *Conclusions*. These results indicate that LIGHT amplification by NF-*κ*B plays a significant role in TLR3 involved hepatitis and points LIGHT to be a potential drug target for liver disease therapy.

## 1. Introduction

LIGHT, a member of the tumor necrosis factor (TNF) superfamily, is a type II transmembrane glycoprotein and a homotrimer expressed on the surface of several immune cells including T cell, granulocytes, monocytes, and dendritic cells [1, 2]. LIGHT binds to herpes virus entry mediator (HVEM) and lymphotoxin-*β* receptor (LT*β*R) to accelerate T cell proliferation and cytokine production. [1, 3, 4]. Previous studies indicate that LIGHT has an important role in inflammatory diseases, such as autoimmune hepatitis, urticaria, asthma, and nonalcoholic fatty liver disease [5–8]. Overexpression of LIGHT in mice also shows immune cell infiltrations in the periportal areas of the liver and has a potential role in the apoptotic cell death of liver cells [9, 10]. Increased serum LIGHT level in people with nonalcoholic fatty liver disease is found to be correlated with liver damage [7]. In addition, LIGHT has been shown to be involved in hepatocyte damage in ConA-induced hepatitis [8]. These studies indicate the crucial role of LIGHT in liver diseases.

TLR3 is a member of TLRs that can recognize double-stranded RNA (dsRNA) from virus genomes or replication intermediates within infected cells [11, 12]. TLR3 also recognizes endogenous dsRNA from apoptosis cells and activates signaling cascades in the absence of infection [13, 14]. TLR3 triggers the recruitment of TIR-domain-containing adapter-inducing interferon-*β* (TRIF) to bind to TNF receptor-associated factor 3 (TRAF3) and receptor interacting protein kinase 1 (RIPK1), leading to the activation of mitogen-activated protein kinase (MAPK), nuclear factor-*κ*B (NF-*κ*B), c-Jun N-terminal Kinase (JNK), and IFN-regulatory factor 3 (IRF3) [11, 15]. A major consequence of these signaling activations is the induction of inflammatory cytokines, which are involved in pathogenesis during the innate and adaptive immune responses [16, 17]. Growing evidences suggest that TLR3 plays an important role in the pathogenesis of liver diseases [18–20]. However, the cellular and molecular mechanisms of TLR3 signaling in liver diseases remain elusive.

In this study, we explored the potential pathogenic role of LIGHT in poly(I:C)-induced acute hepatitis mouse model. We found that LIGHT was upregulated during the course of poly(I:C)-induced acute hepatitis in a NF-*κ*B dependent manner and blocking LIGHT pathway ameliorated liver injury. These results indicate that LIGHT is an important contributor to TLR3 pathway-induced acute hepatitis.

## 2. Methods

### 2.1. Mice and Ethics Statement

Female BALB/c mice (6–8 weeks old) were obtained from Vital River Lab Animal Technology (Beijing, China). The mice were housed in an animal facility at a humidity of 40-60% and a temperature of 22-24°C with a 12 h alternating light and dark cycle. The mice were housed in a standard polypropylene cage with stainless steel top grill having facilities for SPF food and water. These measures were to ensure the mice did not have any unexpected deaths. All research staff was provided for training in animal care or handling and the animal experiments were carried out in strict accordance with the recommendations in the Guide for the Care and Use of Laboratory Animals of the National Institutes of Health and all procedures were also approved by the Animal Care and Use Committee of Shenzhen Institutes of Advanced Technology, Chinese Academy of Sciences (Approval IDs: SCXY 2017-0223). All efforts were made to minimize the suffering and distress of mice. At the end of the experiment, mice were euthanized by the cervical dislocation method.

### 2.2. Preparation of Recombinant HVEM-Fc Fusion Protein

To generate soluble recombinant HVEM–Fc fusion protein, cDNAs encoding human HVEM and human IgG1 were amplified from the RNA of Raji cell by PCR using specific primers (HVEM-Forward: CGAAGCTT CTGCCGTCCTGCAAGGAGG and HVEM-Reverse: CCCGAATTC TACCCAGTGGGAGCTGCTGGT; FC-Forward: GGGAATTCGAGCCCAAATCTTGTGACAAACT and FC-Reverse: CCCTCGAGACCCGGAGACAGGGAGAGGCTCT). The PCR products were isolated as a 0.51-kb HindIII/EcoRI fragment of HVEM and a 0.69-kb EcoRI/XhoI fragment of FC, respectively. The two DNA fragments were then ligated into PCDNA3.1 vector containing the human CMV promoter. Stable cell lines expressing the HVEM-Fc fusion protein were established by transfecting of CHO cells with pcDNA3.1-HVEM-Fc plasmid using Lipofectamine 2000 (Invitrogen) following the manufacturer's instructions. Transfected cells were selected and maintained in G418. The recombinant HVEM-Fc protein was purified from culture supernatants of CHO cells using protein A column [21].

### 2.3. Experimental Protocol

Six to eight-week-old female BALB/c mice were randomly divided into four groups with each group having 8 mice. Because poly(I:C)-induced liver injury is relatively mild, we sensitized mice with D-galactosamine (D-GalN) to the effect of poly(I:C), which activates TLR3 signaling to trigger acute liver inflammation and massive liver damage [22, 23]. For the induction of acute hepatitis, mice were injected i.v. with poly(I:C) (1.5 *μ*g/mouse) (Sigma) and i.p. with D-GalN (10 mg/mouse) (Sigma). The control group was injected with PBS. For blockade experiments, recombinant HVEM-Fc protein was administered by i.p with 100 *μ*g/mouse 1 h before Poly(I:C) and D-GalN injection. For the inhibition of TLR3 downstream signaling pathways, mice were injected i.p. with NF-*κ*B inhibitor (BAY-11-7082,6 mg/kg) (Beyotime, China) 1 h before injecting i.v. with poly(I:C) and i.p. with D-GalN. Blood collection was performed under anesthesia through orbital plexus bleeding from each group at 8, 16, and 24 hours after poly(I:C) and D-GalN administration. Serum ALT levels were measured by a transaminase test kit (Sigma) according to the manufacturer's instruction. For survival studies, mice were monitored at least every 4 hours for 24 hours after a lethal dose of poly(I:C) (2.5 *μ*g/mouse) and D-GalN(10 mg/mouse) injection. Animals used in the experiments were sacrificed for liver removal. Humane endpoints were used in the experiments to analyze molecular parameters within 24 hours after acute hepatitis induction and any criteria includes greater than 20% reduction in body weight, any of the previously mentioned signs of distress that could not be remedied by the veterinary staff, or at the recommendation of the veterinarian. Once animals reached endpoint criteria, the amount of time elapsed before euthanasia was 5-8 min. The animals enrolled in the study were used 184 mice and euthanized 173 mice, and 11 mice died due to acute hepatitis induced by a lethal dose of poly(I:C) and D-GalN injection for survival study. Animals were euthanized at the end of the experiments by the cervical dislocation method, following the guidelines of the Institutional Animal Ethics Committee.

### 2.4. H&E and TUNEL

Mouse liver tissues were fixed in 10% buffered formalin and embedded in paraffin. Tissue sections were cut and stained with hematoxylin and eosin (H&E) or TUNEL (Promega, USA) to observe the level of inflammation and tissue damage by light microscope or fluorescence microscope [24].

### 2.5. RNA Isolation and RT-PCR Analysis

Liver tissues at 0, 2, 4, 6, and 8 hours after the poly(I:C) and D-GalN injection were collected, and total RNA from livers was isolated using an EASYspin Plus RNA Extraction Kit (Aidlab, China) according to the manufacturer's instruction. The total RNA was used for cDNA synthesis by reverse transcription using a PrimeScript RT reagent kit (TaKaRa, China). The specific primers for mouse-LIGHT (sense-5′GGCTGGTTTCTCCTGAGACTG, antisense-5′TGATACGTCAAGCCCCTCAAG) were designed and synthesized by Sangon Company (Shanghai, China). The quantitative real-time PCR was performed using a Light Cycler 480 (Roche, USA). The PCR was performed in a 20 *μ*L volume containing 1 *μ*L cDNA, 10 *μ*L 2 × SYBR Green Premix Ex Taq (TaKaRa), and 0.4 *μ*m each gene-specific primer. Thermal cycling parameters were as follows: 94°C for 2 min, 40 cycles of 94°C for 20 s, 55°C for 20 s, and 72°C for 20 s, followed by one cycle of 95°C for 30 s, 60°C for 30 s, and 95°C for 30 s. The final step was done to obtain a melt curve for the PCR products to determine the specificity of the amplification. All samples were carried out in triplicate on the same plate, and GAPDH was utilized as the reference gene. Expression levels of genes were calculated relative to the expression of the GAPDH. Mouse-GAPDH primers are sense-5′GGTCGGTGTGAACGGATTTGG and antisense-5′CCGTGAGTGGAGTCATACTGGAA) [24].

### 2.6. Western Blot Analysis

Total protein was isolated from livers at 0, 6, 12, 18, and 24 hours after the poly(I:C) injection. Protein lysate was prepared according to standard protocols. Sixty *μ*g of total protein was mixed with loading buffer, boiled and subjected to sodium dodecyl sulfate–polyacrylamide gel electrophoresis (SDS-PAGE). The protein was transferred onto PVDF membranes. Nonspecific binding was blocked with 5% nonfat milk for 2 h at room temperature or overnight at 4°C. The membranes were incubated with different mAbs for 1 h at room temperature. Membranes were washed with PBST (PBS containing 0.1% Tween 20) three times and incubated with a 1 : 5000 dilution of horseradish peroxidase-conjugated secondary Abs at room temperature and were then washed with PBST three times for 15 min each. The following antibodies were used: TNFSF14 (LIGHT) (Biorbyt, orb13725,), *β*-actin (Santa Cruz, sc-47778), and goat-anti-mouse IgG-HRP antibody (Santa Cruz, sc-2005).

### 2.7. Statistical Analysis

The results were analyzed by the Student *t*-test or analysis of variance where appropriate. All data are shown as mean + standard error of the mean (SEM). *p* value of 0.05 was considered to be statistically significant.

## 3. Results

### 3.1. Upregulation of LIGHT Expression in Poly(I:C)-Induced Acute Hepatitis

**Figure 1:**
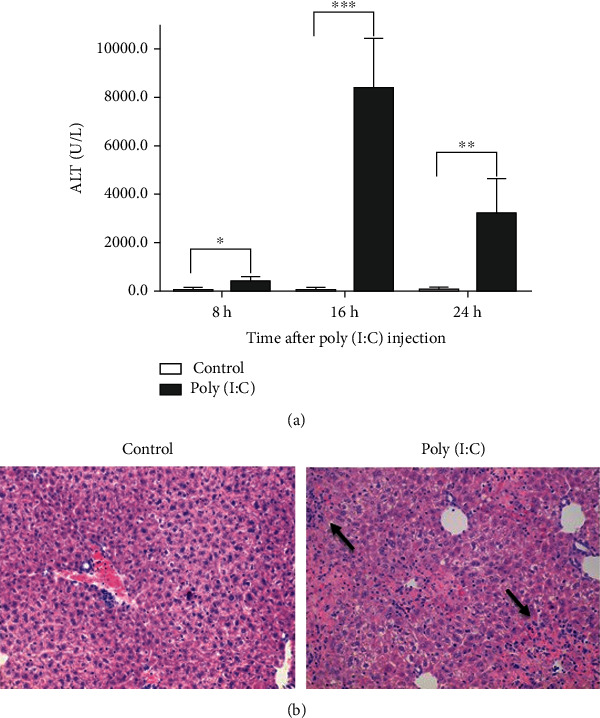
TLR3 signaling triggered acute hepatitis. (a) BALB/c mice were injected with poly(I:C) (1.5 *μ*g/mouse) and D-GalN(10 mg/mouse). At the 8 h, 16 h, and 24 h time points, serum ALT was measured. The figure shows the results of one representative experiment with eight mice per group, and data are shown as mean + SEM; ^∗^*p* < 0.05, ^∗∗^*p* < 0.01, ^∗∗∗^*p* < 0.001. (b) The mice were sacrificed at 24 h after treatment with saline or poly(I:C). Liver sections were stained with H&E staining (size bar represents 50 *μ*m). Arrows indicate necrotic areas with accumulation of mononuclear cells.

**Figure 2:**
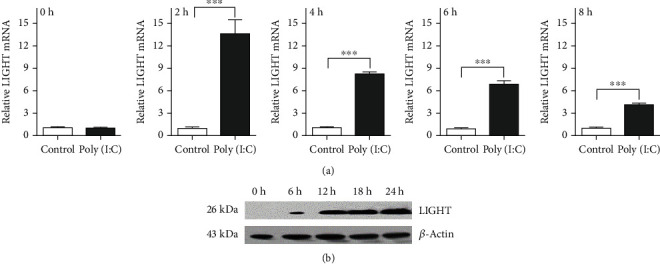
Expression of LIGHT induced by poly(I:C). (a) At the indicated time point, the mice were sacrificed and total RNA was extracted from the liver. Expression of LIGHT mRNA was measured by real-time PCR, and data are shown as mean ± SEM; ^∗∗∗^*p* < 0.001. (b) Expression LIGHT protein was analyzed by western blot at 0 h, 6 h, 12 h, 18 h, and 24 h after poly(I:C) injection.

To investigate whether LIGHT is involved in TLR3-triggered liver diseases, we employed a mouse acute hepatitis model induced by poly(I:C). After challenges with poly(I:C) and D-GalN, mice developed acute hepatitis, indicating by generation of large amount of alanine aminotransferase (ALT) in the serum (Figure 1(a)) and liver damage (Figure 1(b)). Expression of LIGHT mRNA and protein in the livers of the mice was monitored following poly(I:C) injection. Real-time PCR analysis showed that LIGHT mRNA expression was upregulated, which peaked at 2 hours after poly(I:C) injection and gradually declined afterwards (Figure 2(a)). Western blot showed that the expression of LIGHT protein was undetectable in untreated mice, but was induced by poly(I:C) injection, which was detected at 6 hours and continued to increase (Figure 2(b)). The results indicated the expression of LIGHT was induced in the early phase of poly(I:C)-induced acute hepatitis.

### 3.2. Blockade of LIGHT Pathway Using Recombinant HVEM-Fc Fusion Protein Ameliorates Hepatitis Triggered by Poly(I:C)

**Figure 3:**
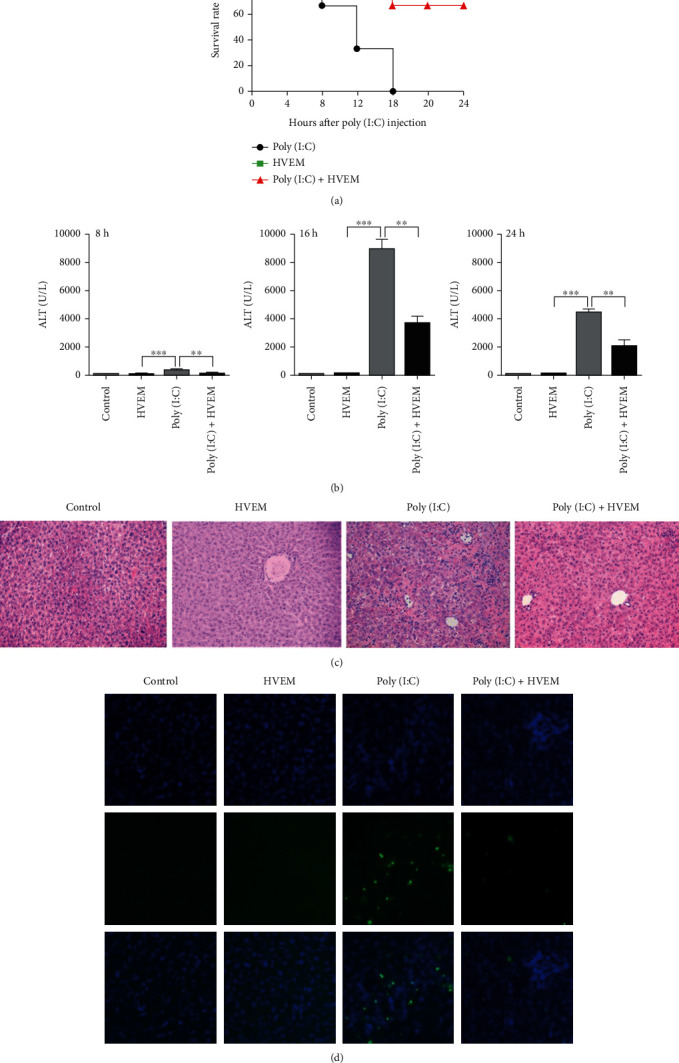
Blockade of LIGHT with recombinant HVEM-Fc fusion protein ameliorates liver injury induced with poly(I:C). (a) BALB/c mice were pretreated with recombinant HVEM-Fc protein (100 *μ*g/mouse) one hour before a lethal dose of poly(I:C) (2.5 *μ*g/mouse) and D-GalN (10 mg/mouse) injection (*n* = 8) or poly(I:C) and D-GalN (*n* = 8) only. The survival of mice was monitored. (b) BALB/c mice were pretreated with recombinant HVEM-Fc protein (100 *μ*g/mouse) one hour before a sublethal dose of poly(I:C) and D-GalN injection (*n* = 8) or poly(I:C) and D-GalN (*n* = 8) only. At 8, 16, and 24 h time point, serum ALT levels were measured and data are shown as mean + SEM; ^∗∗^*p* < 0.01, ^∗∗∗^*p* < 0.001. Mice were sacrificed at 24 h and the liver tissues were fixed and stained with H&E (size bar represents 50 *μ*m) (c) and TUNEL staining (size bar represents 20 *μ*m) (d).

To investigate whether LIGHT plays a role in the pathogenesis of TLR3-triggered acute hepatitis, we blocked the LIGHT signaling pathway with recombinant HVEM-Fc fusion protein, which blocks interaction of LIGHT with its receptor HVEM. Mice were pretreated with recombinant HVEM-Fc fusion protein for one hour before injections with a lethal dose or a sublethal dose of poly(I:C). Twenty-four hours after injections with a lethal dose of poly(I:C), 100% of mice without recombinant HVEM-Fc fusion protein treatment died of acute hepatitis, but more than 80% of mice that received recombinant HVEM-Fc fusion protein survived (Figure 3(a)). We also measured serum ALT activity of the mice receiving sublethal poly(I:C) injection at 8 h, 16 h, and 24 h after injection, and observed that serum ALT levels induced by poly(I:C) were significantly reduced in the mice pretreated with recombinant HVEM-Fc fusion protein compared with those of mice receiving no recombinant HVEM-Fc fusion protein pretreatment (Figure 3(b)). Liver sections from recombinant HVEM-Fc fusion protein treated and untreated mice showed that blockade of LIGHT markedly reduced necrosis and apoptosis in the livers (Figures 3(c) and 3(d)). Taken together, these findings demonstrate that LIGHT plays an important role in the initiation and development of TLR3-triggered hepatitis.

### 3.3. LIGHT Upregulation in Poly(I:C)-Induced Acute Hepatitis Depends on NF-*κ*B Signaling

**Figure 4:**
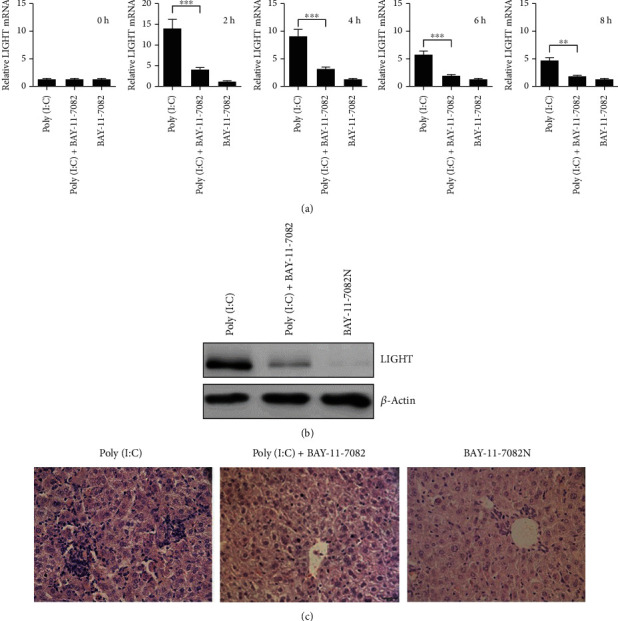
TLR3-induced LIGHT expression through activation of NF-*κ*B. (a) BALB/c mice were injected with poly(I:C) (1.5 *μ*g/mouse) and D-GalN(10 mg/mouse). Mice were pretreated with NF-*κ*B inhibitor (BAY-11-7082) one hour before poly(I:C) injection. The mice were sacrificed and total RNA was extracted from the livers. Expression of LIGHT mRNA was measured by real-time PCR at the indicated time points, and data are shown as mean + SEM; ∗∗*p* < 0.01, ∗∗∗*p* < 0.001. (b) Expression LIGHT protein was analyzed by western blot at 12 h after poly(I:C) injection. (c) Mice were sacrificed at 24 h, the liver tissues were fixed and stained with H&E (size bar represents 20 *μ*m).

NF-*κ*B is a master transcription factor involved in various immune responses and its activation is a critical downstream event of TLR3 stimulation by poly(I:C) treatment. We considered the possibility that poly(I:C)-induced LIGHT upregulation is mediated by NF-*κ*B and examined whether inhibition of NF-*κ*B affects LIGHT upregulation induced by poly(I:C). Mice were pretreated with the NF-*κ*B inhibitor BAY-11-7082 for one hour before poly(I:C) injection. Real-time PCR and western blot analysis showed that treatment with the NF-*κ*B inhibitor significantly reduced expressions of LIGHT mRNA and protein (Figures 4(a) and 4(b)). H&E staining revealed that blocking NF-*κ*B signaling also eased liver injury (Figure 4(c)). These results demonstrate that LIGHT expression is mediated by the NF-*κ*B signaling pathway in TLR3-triggered acute hepatitis.

## 4. Discussion

In the present study, we examined the role of LIGHT in poly(I:C)-induced hepatitis, and revealed that LIGHT was upregulated by NF-*κ*B following poly(I:C) injection and inhibition of LIGHT expression greatly reduced poly(I:C)-induced liver injury. Our study indicates that LIGHT plays an essential role in the pathogenesis of TLR3 signaling-triggered liver injury. The results suggest that breaking LIGHT signaling events may have therapeutic benefit in treating TLR3-mediated hepatitis.

In clinical situations, the enhanced expression of LIGHT in serum of patients with nonalcoholic fatty liver disease, asthma, and diabetes has been detectable [5, 25, 26], which indicates that LIGHT has pathogenic effects in various inflammatory diseases. During the course of experimental acute hepatitis, activation of TLR3 by poly(I:C) led to an enhanced expression of LIGHT in the early phase of hepatitis, and blockade of LIGHT/HVEM pathways with recombinant HVEM-Fc protein significantly reduced serum ALT levels and inhibited liver necrosis and apoptosis in poly(I:C)-induced acute hepatitis.

TLR3 is considered to have a major role in liver diseases [19, 27] and is required for recognizing double-stranded RNA from virus genomes or replication intermediates within infected cells. This includes HCV, HBV, and endogenous dsRNA from apoptosis cells which activate TLR3 signaling in the absence of infection [13, 14, 28, 29]. Activation of TLR3 triggered by its ligand poly(I:C) initiates immune responses with the production of inflammatory cytokines, such as TNF and IFN-*γ* [22, 23]. These molecules participate in inducing the production of inflammatory cytokines and activation of immune cells in the liver and cause liver injury [19, 20]. Our results indicated that LIGHT is overexpressed following TLR3 activation by poly(I:C) and contributes greatly to liver injury.

It has been reported that activation of hepatic NK cells and Kupffer cells triggered by TLR3 initiates the liver injury in poly(I:C)-induced hepatitis model [22]. Previously, study indicated that LIGHT is constitutively expressed on T and NK cells [30]. LIGHT-HVEM engagement plays an important immunomodulatory role in stimulatory T cell interactions, and delivers a costimulatory signal augmenting proinflammatory cytokine production. The study demonstrated NF-*κ*B activation occurred in response to stimulation by ConA earlier than the increases of proinflammatory cytokine production in ConA-induced hepatitis [31]. Cytokines that are stimulated by NF-*κ*B can also directly activate the NF-*κ*B pathway, thus establishing a positive autoregulatory loop that can amplify the inflammatory response [32]. Expression of LIGHT depends on NF-*κ*B activation in TLR3-mediated hepatitis. LIGHT signaling pathway is also involved in activation of NF-*κ*B [33], suggesting LIGHT may be a part of an autocrine-paracrine loop.

Like other TNF superfamily molecules, such as TNF, FasL, and TRIAL, which have been recognized as inflammatory cytokines causing hepatic death and inflammation [34, 35], LIGHT also mediates pathogenic effects in liver diseases [8, 10, 36]. We have previously demonstrated that LIGHT induces immune responses and liver injury in ConA-induced hepatitis. After the injection of a lethal dose of ConA, more than 90% of LIGHT-deficient mice survived indefinitely with significantly lower levels of ALT, whereas 80% of wild type mice died of acute hepatitis [8]. Administration of lymphtoxin-*β* receptor (LT*β*R)-Ig fusion protein protected mice from ConA-induced liver injury through blocking LIGHT signaling [21]. Blockade of any of these ligands described above significantly ameliorates hepatitis. They likely play nonredundant roles and deficiency in one may not be fully compensated for by the others. Enhanced expression of LIGHT is detected in patients with hepatitis C virus infection and Nonalcoholic Fatty Liver Disease [7, 37], indicating that LIGHT may indeed play a role in human hepatitis. We envision that blocking LIGHT signaling may be a promising therapeutic strategy for hepatitis.

## 5. Conclusion

In conclusion, our results uncovered a role of LIGHT in poly(I:C)-induced acute hepatitis, which may lead to new therapeutic strategies targeting this molecule in treating liver diseases and shed new insights into the pathogenesis for TLR3-triggered inflammation diseases.

## Data Availability

The datasets supporting the conclusions of this article are included within the article.

## Abbreviations

ALT: Alanine aminotransferase
ConA: Concanavalin A
dsRNA: Double-stranded RNA
D-GalN: D-galactosamine
HBV: Hepatitis B virus
HCV: Hepatitis C virus
HVEM: The herpes virus entry mediator
H&E: Hematoxylin and eosin
IFN-*γ*: Interferon gamma
JNK: C-Jun N-terminal kinase
LIGHT: Tumor necrosis factor superfamily 14
LT*β*R: Lymphotoxin-*β* receptor
MAPK: Mitogen-activated protein kinase
Poly(I:C): Polyinosinic-polycytidylic acid
TLR: Toll-like receptor
TRAF3: Tumor-necrosis factor receptor associated factor-3
TRAIL: TNF related apoptosis inducing ligand.
